# Unraveling the role of miRNAs as biomarkers in Chagas cardiomyopathy: Insights into molecular pathophysiology

**DOI:** 10.1371/journal.pntd.0011865

**Published:** 2024-02-01

**Authors:** Heriks Gomes Ribeiro, Ony Araújo Galdino, Karla Simone Costa de Souza, Antonia Pereira Rosa Neta, Hui Tzu Lin-Wang, Edecio Cunha-Neto, Adriana Augusto de Rezende, Vivian Nogueira Silbiger

**Affiliations:** 1 Department of Clinical and Toxicological Analysis, Federal University of Rio Grande do Norte, Natal, Brazil; 2 Molecular Biology Laboratory, Dante Pazzanese Institute of Cardiology, São Paulo, Brazil; 3 Laboratory of Immunology, Heart Institute (InCor), University of São Paulo, School of Medicine, São Paulo, Brazil; 4 Division of Clinical Immunology and Allergy, University of São Paulo, School of Medicine, São Paulo, Brazil; 5 Institute for Investigation in Immunology (iii), INCT, São Paulo, Brazil; 6 Translational Medicine, The Hospital for Sick Children, Toronto, Ontario, Canada; Centro de Pesquisa Gonçalo Moniz-FIOCRUZ/BA, BRAZIL

## Abstract

**Background:**

Chagas cardiomyopathy (ChCM) is a severe form of Chagas disease and a major cause of cardiovascular morbidity and mortality. The dysregulation of the immune response leads to cardiac remodeling and functional disruptions, resulting in life-threatening complications. Conventional diagnostic methods have limitations, and therapeutic response evaluation is challenging. MicroRNAs (miRNAs), important regulators of gene expression, show potential as biomarkers for diagnosis and prognosis.

**Aim:**

This review aims to summarize experimental findings on miRNA expression in ChCM and explore the potential of these miRNAs as biomarkers of Chagas disease.

**Methods:**

The search was conducted in the US National Library of Medicine MEDLINE/PubMed public database using the terms “Chagas cardiomyopathy” OR “Chagas disease” AND “microRNA” OR “miRNA” OR “miR.” Additionally, bioinformatics analysis was performed to investigate miRNA-target interactions and explore enrichment pathways of gene ontology biological processes and molecular functions.

**Results:**

The miR-21, miR-146b, miR-146a, and miR-155 consistently exhibited up-regulation, whereas miR-145 was down-regulated in ChCM. These specific miRNAs have been linked to fibrosis, immune response, and inflammatory processes in heart tissue. Moreover, the findings from various studies indicate that these miRNAs have the potential as biomarkers for the disease and could be targeted in therapeutic strategies for ChCM.

**Conclusion:**

In this review, we point out miR-21, miR-146b, miR-146a, miR-155, and miR-145-5p role in the complex mechanisms of ChCM. These miRNAs have been shown as potential biomarkers for precise diagnosis, reliable prognostic evaluation, and effective treatment strategies in the ChCM.

## Introduction

Chagas disease (ChD) is caused by the parasite *Trypanosoma cruzi* and affects millions worldwide, primarily in Latin American countries [1]. While many infected individuals remain asymptomatic during the chronic phase, around 30% develop the cardiac form known as Chagas cardiomyopathy (ChCM), which is the most severe form of ChD and is a major cause of cardiovascular-related morbidity and mortality in endemic areas [2,3]. The dysregulation of the immune response is believed to trigger cardiac remodeling, leading to hypertrophy, fibrosis, edema, and disruptions in heart function, which can result in life-threatening arrhythmias, heart failure, thromboembolism, and sudden death [3,4].

Despite being described over a century ago, many aspects of ChD remain unresolved. Currently, conventional diagnostic and prognostic methods for ChCM primarily detect functional and structural changes in the heart [5]. Consequently, clinical and therapeutic interventions rely on the onset and progression of cardiac damage [6]. Another challenge is accurately evaluating the therapeutic response to *T*. *cruzi*, which still lacks satisfactory methods, raising concerns about the clinical management of patients [7]. Several studies have proposed biological molecules (immunological, biochemical, and molecular or nucleic acid-based biomarkers) as biomarkers for monitoring and treatment of patients with ChD, but their practical implementation has been limited, indicating there are still gaps in this area of knowledge [8–10].

Nevertheless, over the years, research has shed light on the role of microRNAs (miRNAs) in the pathogenesis and progression of ChCM [11–14]. These miRNAs are endogenous, conserved, small (approximately 22 nucleotides) non-coding RNA molecules that have critical roles in regulating gene expression at the posttranscriptional level. They can bind to complementary messenger RNA (mRNA) sites inhibiting protein translation or promoting mRNA decay [15,16].

Notably, miRNAs are involved in the regulation of major cellular activities, such as metabolism, differentiation, proliferation, as well as apoptosis [17,18] and its amount has been shown to be dysregulated in different disorders, including cardiac conditions, bacterial and viral infections, and parasitic infections [16,19]. These functional miRNAs can be detected in various body fluids, such as whole blood, serum, and plasma, making them potential biomarkers for the diagnosis and prognosis of the disease [19]. Further, although the exact mechanism by which miRNAs are involved in ChCM pathophysiology remains unclear, studies have revealed that the biogenesis of miRNAs implicated in cardiac physiology is affected by *T*. *cruzi* infection, and thus miRNAs could potentially serve as prospective biomarkers for ChCM [11–14,20–22].

In the context of ChCM, various studies have explored miRNAs’ expression profiles in patients and identified specific miRNAs that are dysregulated in cardiac tissues [11–14]. These findings suggest that miRNAs could be potential biomarkers for diagnosing ChCM and monitoring disease progression [12,20]. Furthermore, experimental studies have demonstrated the involvement of specific miRNAs in regulating key molecular pathways implicated in ChCM, including inflammation, fibrosis, and apoptosis [12–14,21,22]. Consequently, targeting these miRNAs has shown promise as a potential therapeutic strategy for ChCM, with preclinical studies highlighting the beneficial effects of miRNA-based interventions in animal models of the disease [22,23].

Therefore, this review encompasses studies that have elucidated the involvement of miRNAs in the mechanisms underlying ChCM. Furthermore, it explores their function in molecular pathways involved in ChD and the potential application of miRNAs as biomarkers for accurate diagnosis, prognostic assessment, and therapeutic interventions of ChCM.

## Methods

Authors HGR and OAG conducted a comprehensive literature search of all original articles published up until October 15, 2023, to identify potential miRNA biomarkers in ChCM. The search was performed using the US National Library of Medicine MEDLINE/PubMed public database and included the terms “Chagas cardiomyopathy” OR “Chagas disease” AND “microRNA” OR “miRNA” OR “miR.” Additionally, MeSH terms (“Chagas Cardiomyopathy”[Mesh]) AND “MicroRNAs”[Mesh] were also included.

### Selection and description of studies

**Fig 1:**
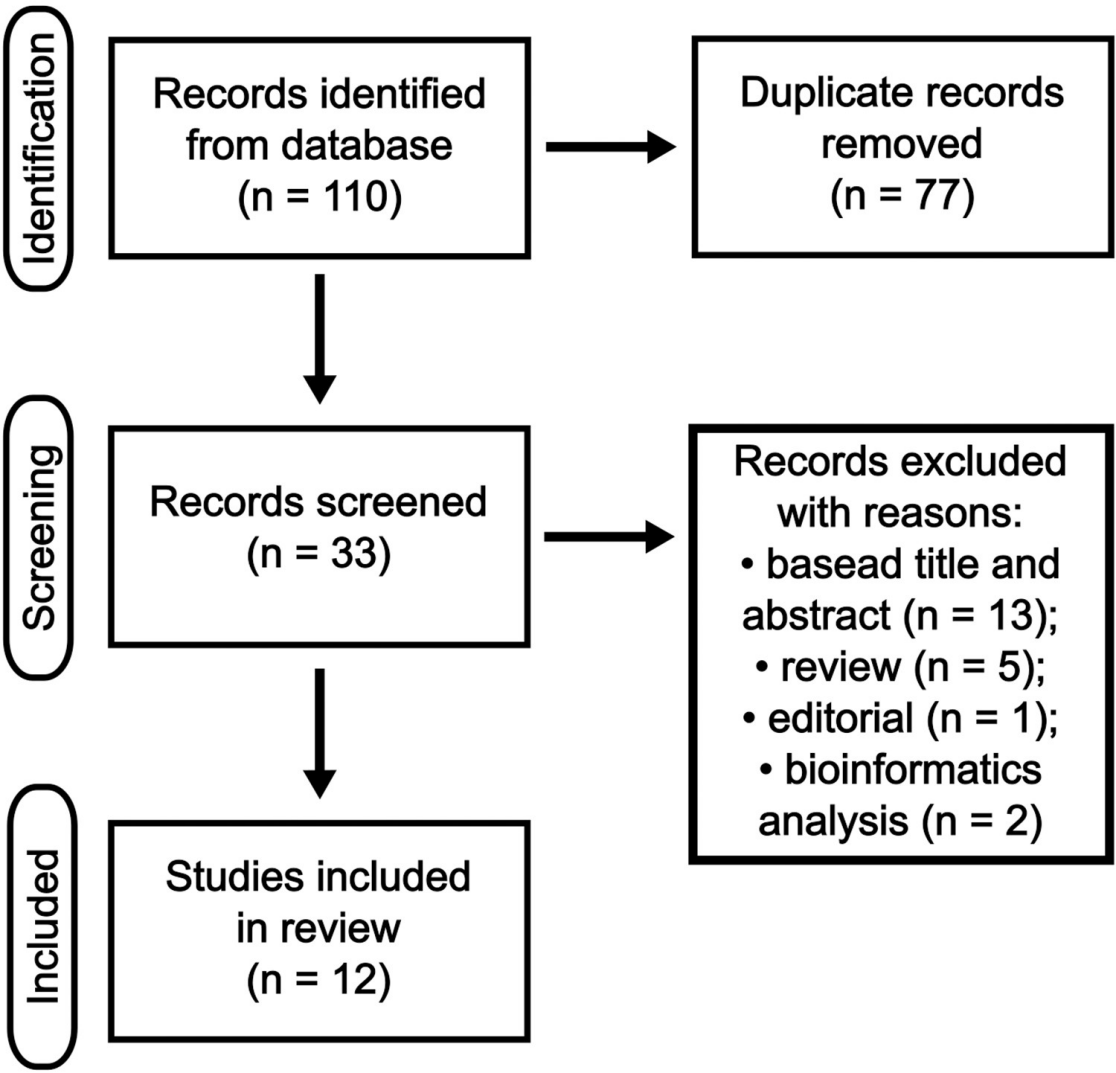
Flow diagram applied to select studies that involve the function of miRNAs with the development, monitoring, or prognosis of Chagas cardiomyopathy.

The studies that investigated the relationship between miRNAs in culture cells, tissue, exosome, serum, plasma, or whole blood in the ChD context or involved in the development, monitoring, or prognosis of ChCM were included in this review. Preliminarily, we screened the title and abstract, articles with replicates and unrelated were discarded. Relevant citations were retrieved as complete manuscripts and assessed for compliance with inclusion and exclusion criteria. In the initial search, a total of 110 articles were identified using 7 different combinations of terms. Of these, 77 duplicates, 5 reviews, 13 that did not specifically address ChCM, 2 that were bioinformatics analyses, and 1 that was an editorial were excluded (Fig 1). Ultimately, 12 articles were chosen for inclusion in this review as they met the predefined inclusion criteria.

**Fig 2:**
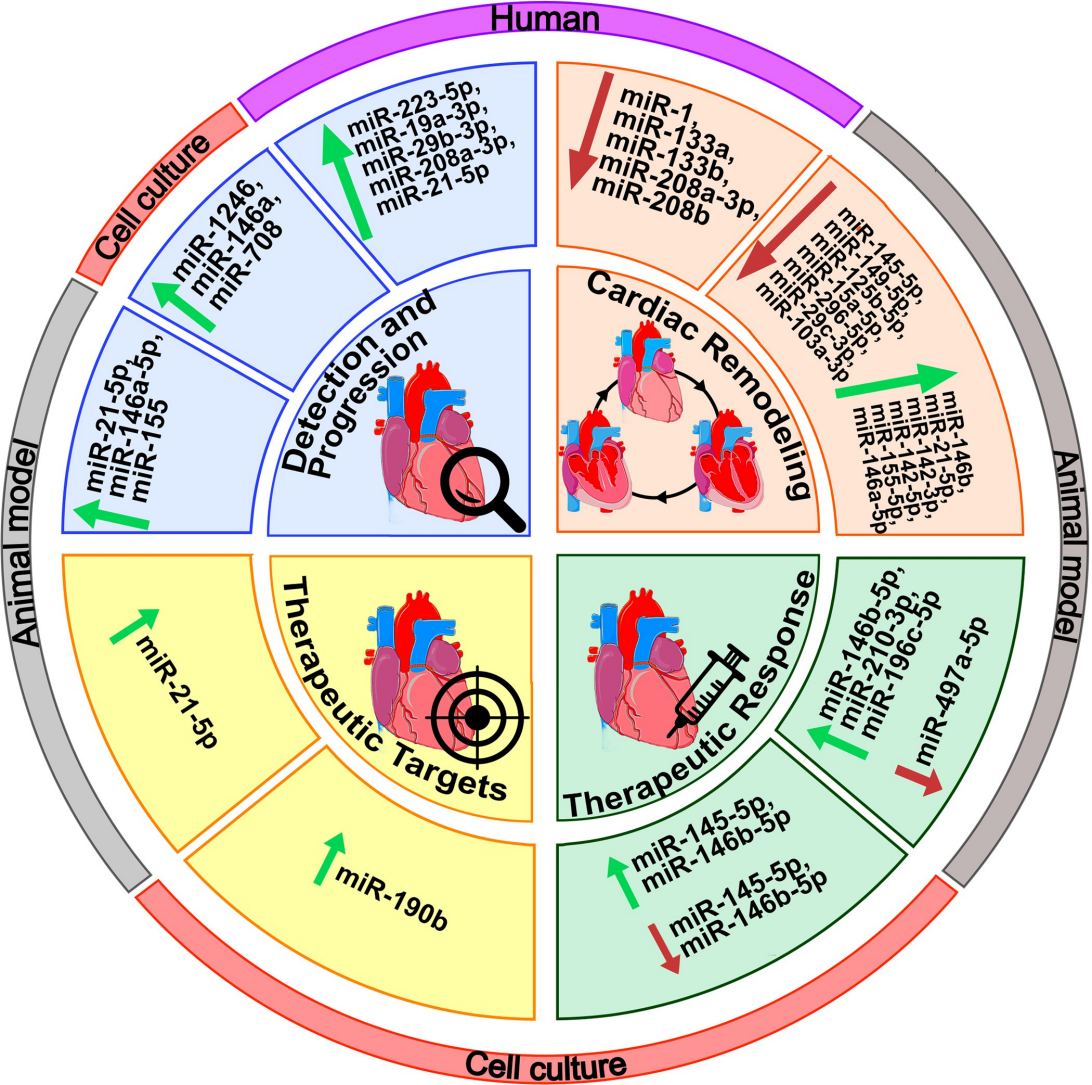
miRNAs were included considering the contexts of the studies and type of samples used. miRNAs were grouped into quadrants according to the context of the studies. The outer edge is color-coded to represent the sample type. The hearts in this figure have been modified from Servier Medical Art [28], licensed under a Creative Common Attribution 3.0 Generic License [29].

Among the 12 experimental articles included in this review, 4 research studies utilized animal models, 5 involved cultured cells, and 6 studies were conducted using patient samples (Fig 2 and Table 1). About the sample types, 7 experiments utilized heart tissue, 4 used serum/plasma, 2 involved H9C2 cells infected, 1 utilized induced pluripotent stem cells-derived cardiomyocytes (iPSC-CM), 1 utilized cardiomyocyte, epithelial cells (HeLa), and macrophages (THP1-derived) from human and 1 used fibroblast cells derived from the cardiac tissue of patients (Table 1). Specifically, concerning the patients assessed in the studies, a population of 218 individuals was included [11,13,14,20,21,24]. This cohort consisted of 146 patients diagnosed with ChCM, 20 patients with an indeterminate form of ChD, 46 individuals classified as control subjects without ChCM, and 6 patients with idiopathic dilated cardiomyopathy (DCM) (Table 1). Further, heterogeneity was observed regarding the severity of the ChCM screened by the studies. For instance, Laugier and colleagues [20] focus on patients in the advanced stage of the disease who met the criteria for heart transplantation, while Gómez-Ochoa and colleagues [11] specifically included ChCM patients with reduced left ventricular ejection fraction (LVEF) (LVEF ≤ 40%).

**Table 1:** Review of subject, sample type, miRNA isolation, and miRNAs studied.

Authors	Subject	Sample type	Method of RNA isolation	Total miRNA study	Up-regulated	Down-regulated
Silva Grijó Farani and colleagues (2023) [25]	C57BL/6 mice model–groups non-infected, vehicle, Bz and Bz + PTX (*n* = 3 per group)	Total heart tissue	mirVana miRNA Isolation Kit (Life Technologies)	752	**miR-146b-5p**, mir-210-3p, mir-196c-5p	mir-497a-5p
Gómez-Ochoa and colleagues (2022) [11]	Human—ChCM (*n* = 74)	Serum	miRNeasy Serum/Plasma Advanced Kit (Qiagen)	6	miR-223-5p	-
Nonaka and colleagues (2021) [21]	C57Bl/6 mice model–groups ChCM (*n* = 8), CONT (*n* = 4) and human ChCM (*n* = 7)	Heart tissue animal model and human serum	miRCURY RNA isolation kit (Exiqon)	88	**miR-21-5p**	-
Farani and colleagues (2022) [23]	-	H9C2 cells	mirVana miRNA Isolation Kit (Life Technologies)	3	**miR-145-5p** and **miR-146b-5p** - without treatment	**miR-145-5p** and **miR-146b-5p** in Bz + PTX treatment
Ballinas-Verdugo and colleagues (2021) [12]	CD1 mice model—groups case (*n* = 20) and CONT (*n* = 20)	Plasma, heart tissue, microvesicles	miRNeasy Serum/Plasma Advanced Kit/exoRNeasy Serum/Plasma midi kit (Qiagen)	3	miRNAs **miR-21-5p**, **miR-146a-5p** and **miR-155** (acutely and chronically)/**miR-146a-5p** (each sample in both phases)	-
Laugier and colleagues (2020) [20]	Human—groups ChCM (*n* = 8) and CONT (*n* = 4)	Left ventricular heart tissue	RNeasy Mini Kit (Qiagen) adapted with Trizol	754	**miR-155-5p** and **miR-146a-5p**	hsa-miR-125b-5p, hsa-miR-15a-5p, hsa-miR-296-5p, hsa-miR-29c-3p and hsa-miR-103a-3p
Nonaka and colleagues (2019) [13]	Human—groups ChCM (*n* = 28), IF (*n* = 10), HC (*n* = 10) for circulating miRNA and ChCM (*n* = 9), CONT (*n* = 8) for heart tissue	Serum and plasma in exosomes/iPSC-CM and fibroblast from heart tissue	miRCURY Exosome Isolation Kit (Exiqon)/Trizol (Life Technologies)	6	miR-19a-3p, **miR-21-5p**, and miR-29b-3p	-
Navarro and colleagues (2015) [22]	C57BL6 mice model—groups CONT, 15 dpi, 30 dpi, and 45 dpi (*n* = 12 per group)	Left and right heart ventricles	mirVana miRNA Isolation Kit (Life Technologies)	641	**miR-146b, miR-21-5p**, miR-142-3p and miR-142-5p	**miR-145-5p** and miR-149-5p
Ferreira and colleagues (2014) [24]	Human—groups ChCM (*n* = 10), DCM (*n* = 6), and CONT (*n* = 4)	Left ventricular heart tissue	mirVana miRNA Isolation Kit (Life Technologies)	9	-	miR-1, miR-133a-2, miR-133b, **miR-208a-3p**, and 208b (ChCM x CONT); miR-133b, **miR-208a-3p** and miR-208b (DCM x CONT)
Linhares-Lacerda and colleagues (2018) [14]	Human—groups ChCM (*n* = 10), IF (*n* = 10), CONT (*n* = 20)	Plasma	miRNeasy Serum/Plasma Kit (Qiagen)	2	**miR-208a-3p**	-
Monteiro and colleagues (2015) [27]	-	H9C2 cells	Trizol (Life Technologies)	5	miR-190b	-
Rego and colleagues (2023) [26]	-	Cardiomyocytes, HeLa and THP1-derived	Direct-zol RNA MiniPrep kit (Zymo, USA)	43	miR-1246, miR-708-5p and **146a-5p**	-

In bold are the miRNAs that appeared in more than one study included in this review. C57BL/6: mice strain model; Bz: benznidazole; PTX: pentoxifylline; ChCM: Chagas cardiomyopathy; CD1: mice strain model; IF: indeterminate form; dpi: days postinfection; CONT: control; DCM: idiopathic dilated cardiomyopathy; H9C2 cells: H9C2 rat cardiomyoblast cell line; iPSC-CM: induced pluripotent stem cells-derived cardiomyocytes; HeLa: epithelial cells; THP1-derived: macrophages.

Regarding the miRNA isolation which depends on the type of sample under investigation, most studies used commercial kits that employed Spin Column-based isolation technology (Table 1). Considering the assessments of expression level, a total of 2,311 miRNAs were evaluated (Table 1). Silva Grijó Farani and colleagues [25] and Laugier and colleagues [20] utilized comparable commercial miRNA array panels comprising 752 miRNAs from the same manufacturer. Conversely, Nonaka and colleagues (2021) [21] investigated 88 miRNAs, while Navarro and colleagues [22] examined 642 miRNAs using quantitative real-time reverse-transcription PCR (qRT-PCR) arrays. Rego and colleagues [26] used miRNA sequencing. The remaining studies focused on individual candidate miRNAs, analyzing them using qRT-PCR [11–14,23,24,27].

### miRNA-target interaction and enrichment analysis

A bioinformatics approach was performed to demonstrate the relationship between miRNAs and molecular and biological processes that may be related to ChCM. All analyses were performed only with the miRNAs shown in the studies, which were more prevalent and whose nucleotide sequences were conserved among the species used in the experiments and humans. The miRBase database [30] was used to query the miRNA sequences. The selection of targets was carried out at miRDB—MicroRNA Target Prediction Database [31]. Only targets with “Target Score” > = 80 were considered. Gene Ontology (GO) for Biological Processes and Molecular Functions was performed at EnrichR [32], considering terms related to cardiology and immunology. GO processes considered significant had a *p*-value < 0.05. The analysis and plots generated were performed using the software RStudio Version 2023.06.0+421 with Packages ggplot2 Version 3.4.2 [33] and Circlize Version 0.4.15 [34].

### miRNA for detection and progression

Gòmez-Ochoa and colleagues [11] discovered a striking correlation between the expression levels of circulating miR-223-5p by qRT-PCR in serum samples. They correlated both echocardiographic and laboratory biomarkers of myocardial injury in a group of 74 patients with ChCM. Lower levels of miR-223-5p were associated with the worsening of ChCM, potentially attributed to signaling pathways associated with receptor tyrosine kinases. These findings provide insights into the mechanistic role of miR-223-5p in the progression of this condition.

In another study conducted by Ballinas-Verdugo and colleagues [12], the overexpression of inflammatory microRNAs miR-21, miR-146a, and miR-155 were evaluated in heart tissue, plasma, and plasma extracellular vesicles from mice infected with the Mexican *TcI Ninoa* strain. The qRT-PCR was performed to assess miRNAs in the acute and chronic phases of infected mice. The study also identified 23 functional pathways associated with *T*. *cruzi* infection. Furthermore, bioinformatics analysis identified 11 genes, including down-regulated SMAD family member five, which is a mediator in the transforming growth factor-beta (TGF-β) signaling pathway. The researchers emphasized that miR-146a could be a potential noninvasive biomarker for the early detection of ChD.

Nonaka and colleagues (2019) [13] assessed 48 subjects, including individuals with the cardiac form of ChD presenting left ventricular dysfunction, indeterminate individuals without symptoms of ChD, and healthy controls. The qRT-PCR was applied to evaluate miRNA expression in exosomes derived from serum and plasma samples. The findings revealed significant alterations in the expression levels of circulating miRNAs in ChD, some of which correlated with the expression profile in cardiac tissue. Notably, elevated levels of miR-19a-3p, miR-21-5p, and miR-29b-3p in circulation were associated with cardiac injury and the severity of ChD. The authors further validated their finding of miRNAs through in vitro models of cardiac fibrosis using TGF-1-stimulated cardiac fibroblast cells and hypertrophy myocardial model using human-induced pluripotent stem cell-derived cardiomyocytes (hiPSC-CM) stimulated with endothelin-1. Elevated expression of miR-19a-3p, miR-21-5p, miR-29b-3p, and miR-199b-5p was observed in hiPSC-CM, while miR-21-5p was up-regulated in cardiac fibroblasts. These results provide insights into the potential roles of specific miRNAs in the pathogenesis and progression of ChCM.

Linhares-Lacerda and colleagues [14] conducted a miRNA expression level analysis by qRT-PCR using serum samples from 40 patients, including those indeterminate individuals without ChD symptoms and non-infected controls. Interestingly, the elevated levels of circulating miR-208a were identified only in the indeterminate form of ChD. It suggests that circulating miR-208a could be a potential biomarker of ChD when it is under control.

Rego and colleagues [26] conducted a study using cardiomyocytes, epithelial tissue, and macrophages from humans. The cells were infected with *T*. *cruzi*, and analyses were carried out 24 h after infection. miRNA sequencing was conducted, and robust bioinformatics analyses revealed that macrophages were more responsive to infection than other cell types. In the context of results among different tissue types, cardiomyocytes showed a higher degree of infection in the early stages than other cells. As demonstrated in previous studies, miRNA-146a is strongly associated with parasite infection. Furthermore, the study revealed the up-regulation of miR-1246 and miR-708, which have not yet been reported in the context of infection. Functional analyses did not reveal a direct relationship with targets already recognized in previous studies. However, further studies are needed to understand the role of these miRNAs and their potential use as biomarkers in ChCM.

### miRNAs involved in cardiac remodeling

ChCM presents a worse prognosis compared to other heart diseases. In a study by Ferreira and colleagues [24], the differences in miRNA expression profiles between DCM and ChCM were evaluated. Human left ventricular free wall heart tissue samples were obtained from end-stage heart failure patients, including ChCM samples (*n* = 10), DCM samples with negative serology for ChD and ischemic disease (*n* = 6), and control samples from healthy hearts (*n* = 4). Ingenuity Pathways Analysis (IPA) was utilized for the Target Prediction of differentially expressed genes (DEGs) and their relationship with the analyzed miRNAs. Five miRNAs (miR-1, miR-133a-2, miR-133b, miR-208a, and miR-208b) showed differential expression between ChCM samples and controls. Specifically comparing ChCM and DCM, miR-1, miR-133a-2, and miR-208b were down-regulated in ChCM. These miRNAs seem to interact with essential genes involved in the heart’s electrical conduction, such as Serine/Threonine Kinase 1 (AKT), Cyclin D1 (CCND1), Interferon (IFN), Growth hormone, Collagen Type 1, and Nuclear Factor kappa B (NF-κB). Notably, miR-208a and miR-208b showed significant positive associations (*p*-value = 0.0007) when comparing ChCM and controls, indicating their specific roles in heart muscle regulation, particularly GATA Binding Protein 4.

Continuing the pioneering studies published by Ferreira and colleagues [24], Navarro and colleagues [22] investigated the role of miRNAs in disease progression following *T*. *cruzi* infection, which is associated with various cardiovascular disorders. Using a qRT-PCR array, they screened 641 rodent miRNAs in heart samples of mice during acute infection with the *Colombian T*. *cruzi* strain. Seventeen miRNAs were significantly dysregulated at all 3 analyzed time points postinfection. Among these, miR-146b, miR-21, miR-142-3p, and miR-142-5p were up-regulated, while miR-145-5p and miR-149-5p were down-regulated, and their expression correlated with parasitemia and the maximal heart rate-corrected QT (QTc) interval. Interestingly, although this study focused on the acute phase of experimental ChD, some miRNAs (miR-133, miR-208) were found to be down-regulated at 45 days postinfection, consistent with previous reports in the hearts of chronic Chagas patients.

In a study by Laugier and colleagues [20], an integrative genome-wide analysis was conducted to understand the role of miRNAs in the pathophysiology of ChCM. Tissue samples from the left ventricular wall of patients with ChCM undergoing heart transplantation (*n* = 8) and of the organ donors (control group) (*n* = 4) were used for miRNA analysis. Interaction analyses between DEGs and differentially expressed miRNAs (DEMs) revealed relationships in important biological processes related to ChCM, including inflammation, interferon gamma-induced genes, fibrosis, extracellular matrix, and hypertrophy processes. Five miRNAs (hsa-miR-125b-5p, hsa-miR-15a-5p, hsa-miR-296-5p, hsa-miR-29c-3p, and hsa-miR-103a-3p) showed clear correlations in the regulation of these processes, suggesting their potential as targets for further studies and therapeutic candidates in the context of ChCM.

### miRNAs for therapeutic response

The first evidence of miR-145-5p down-regulation and miR-146b-5p up-regulation in acute Chagas’ heart disease was reported by Navarro and colleagues [22]. Building on this, Farani and colleagues [23] conducted a study to explore the effects of interventions with the trypanosomicidal drug Benznidazole (Bz) alone or in combination with Pentoxifylline (PTX) on parasite load and the expression of miR-145-5p and miR-146b-5p. They utilized the H9C2 rat cardiomyoblast cell line infected with the *Colombian T*. *cruzi* strain as a model to investigate the interplay between the parasite and host cells. The Bz at concentrations of 3 μm and 10 μm, 48 h after infection, reduced parasite load but did not affect the levels of miR-145-5p and miR-146b-5p by qRT-PCR analyzed. The addition of PTX did not interfere with the parasite control efficacy induced by Bz. However, the combined treatment of Bz + PTX resulted in decreased levels of both miRNAs, with expression levels like those observed in non-infected H9C2 cells.

Furthermore, using mimic/inhibitor systems for miR-145-5p and miR-146b-5p before infection of H9C2 cells, a reduction in parasite load was observed 72 h postinfection. When the mimic/inhibitor systems were applied 48 h after infection, all systems except the miR-146b-5p inhibitor resulted in a reduction in parasite load. These findings suggest that miR-145-5p and miR-146b-5p may play a role in controlling signaling pathways crucial for interacting with the parasite and host cells [23]. Therefore, further investigation of these miRNAs is warranted as potential biomarkers for parasite control and tools to identify therapeutic adjuvants for etiological treatment in ChD.

A recent study conducted by Silva Grijó Farani and colleagues [25] also investigated the expression profiles of 752 miRNAs by qRT-PCR array in the cardiac tissue of mice with chronic *T*. *cruzi* infection treated with Bz, PTX, or a combination of both (Bz + PTX). After 150 days of infection, the Bz + PTX-treated group showed 58 DEMs associated with key signaling pathways related to cellular growth, tissue development, cardiac fibrosis, damage, and cell death. Similarly, the Bz-treated group exhibited 68 DEMs involved in pathways such as cell cycle, cell death and survival, tissue morphology, and connective tissue function. Notably, the combined therapy restored the overexpression of miR-146b-5p, miR-196c-5p, and miR-210-3p, and the underexpression of miR-497a-5p, which play roles in regulating genes associated with heart damage, cell death, and fibrosis pathways. Further analysis revealed that miR-146b-5p directly targeted the mRNA of gene *IL10* (Interleukin 10), *TNF* (Tumor Necrosis Factor), *MMP9* (Matrix Metallopeptidase 9), *ERK* (Mitogen-Activated Protein Kinase 1), and *JNK1* (Mitogen-Activated Protein Kinase 8). In contrast, miR-196c-5p targeted *ANXA1* (Annexin A1) and *BAK1*, among others. These findings emphasize the complexity of ChCM and the central role of miR-146b-5p in cardiotoxicity networks, which was validated as a potential therapeutic target. The study also demonstrated that the association of Bz and Bz + PTX therapies could reverse the up-regulation of miR-146b-5p in the infected group, highlighting their potential for mitigating ChCM progression.

### miRNAs as therapeutic targets

Nonaka and colleagues (2021) [21] proposed miR-21 silencing as a possible therapy for ChCM. Through the combination of PCR array processes and in silico analyses, they identified that miR-21 was up-regulated in cardiac tissue samples from mice and human serum. In addition, functional studies showed that miR-21 was associated with important regulatory pathways of collagen expression (TGFβ1). Twelve C57Bl/6 mice were used in the locked nucleic acid (LNA)-anti-miR-21 inhibitor promoted efficacy test. Mice were infected by intraperitoneal injection of 1,000 trypomastigotes of the *Colombian T*. *cruzi* strain. Heart samples from chronic chagasic mice (*n* = 8) and non-infected controls (*n* = 4) were evaluated after 6 months. The results showed the reduction of miR-21 expression in the cardiac tissue of mice treated with anti-miR-21 compared to untreated mice.

Another study using H9C2 cells infected with the *T*. *cruzi Berenice* 62 strain and assessed the expression of specific miRNAs (miR-16-5p, miR-let7f-2-3p, miR-26b, miR-3586-3p, miR-190b) by qRT-PCR [27]. Subsequently, luciferase reporter assays were conducted, explicitly targeting miR-190b with a transiently introduced no-miR-190b inhibitor. The results demonstrated a correlation between miRNA-190b and decreased cellular viability rates, achieved by negatively modulating phosphatase and tensin homolog (PTEN) protein expression in *T*. *cruzi*-infected cells. PTEN is a tumor suppressor and negative regulator of PI3K signaling and plays a crucial role in hydrolyzing PIP3 to phosphatidylinositol-4,5-bisphosphate. Previous studies have shown that PTEN is reduced during hypertrophic cardiomyopathy and is associated with remodeling in cardiomyocytes [35]. This study sheds light on the modulation of cellular behavior by parasite infection, ensuring the survival of *T*. *cruzi*. In the study of Monteiro and colleagues [27], the reduced expression of PTEN, favored by the increase in expression levels of 5 miRNAs, including miR-190b, during the early stages of *T*. *cruzi* Be-62 infection, underscores its involvement in PTEN regulation and calls for further investigation.

### miRNAs-targets in ChCM

miR-1 and miR-133 regulate the AKT gene, part of the RTK communication pathway. This pathway has been associated with cardiac hypertrophy in the chronic phase of the disease [24]. Within the same pathway, it has been reported that miR-223 regulates the MAPK gene, while miR-208a, miR-208b, and miR-133b target NF-κB. These genes have been identified as immune response modulators [20,24]. Furthermore, NF-κB has also been linked to the interferon-gamma pathway, frequently mentioned in conjunction with mitochondrial dysfunction in ChCM [20,36]. miR-146b, 208a, and 15b are associated with regulating genes from the SMAD family (2/3/5), which have been reported in the TGF-β pathway. This pathway is consistently referred to as a means of host cell invasion by *T*. *cruzi* [12,14]. Some key genes are connected to conduction disorders that can alter cardiac electrophysiology. The CACNA1C and KCNA1 genes encode a subunit of a calcium channel and a subunit of a potassium channel, respectively. The CACNA1C gene is regulated by miR-149, while miR-21-5p and miR-145-5p are involved in KCNA1 regulation. The GJA5 gene, which encodes Gap Junction Protein Alpha 5, is regulated by miR-208a and miR-145-5p [22]. Furthermore, miR-1 has also been reported to regulate the SERCA2a gene, which encodes the sarcoplasmic/endoplasmic reticulum Ca2+-ATPase responsible for transporting calcium from the cytosol to the sarcoplasmic reticulum. This pathway is associated with the progression of dilated cardiomyopathy [24]. These mechanisms and pathways can potentially explain important aspects of the development and progression of ChCM.

**Fig 3:**
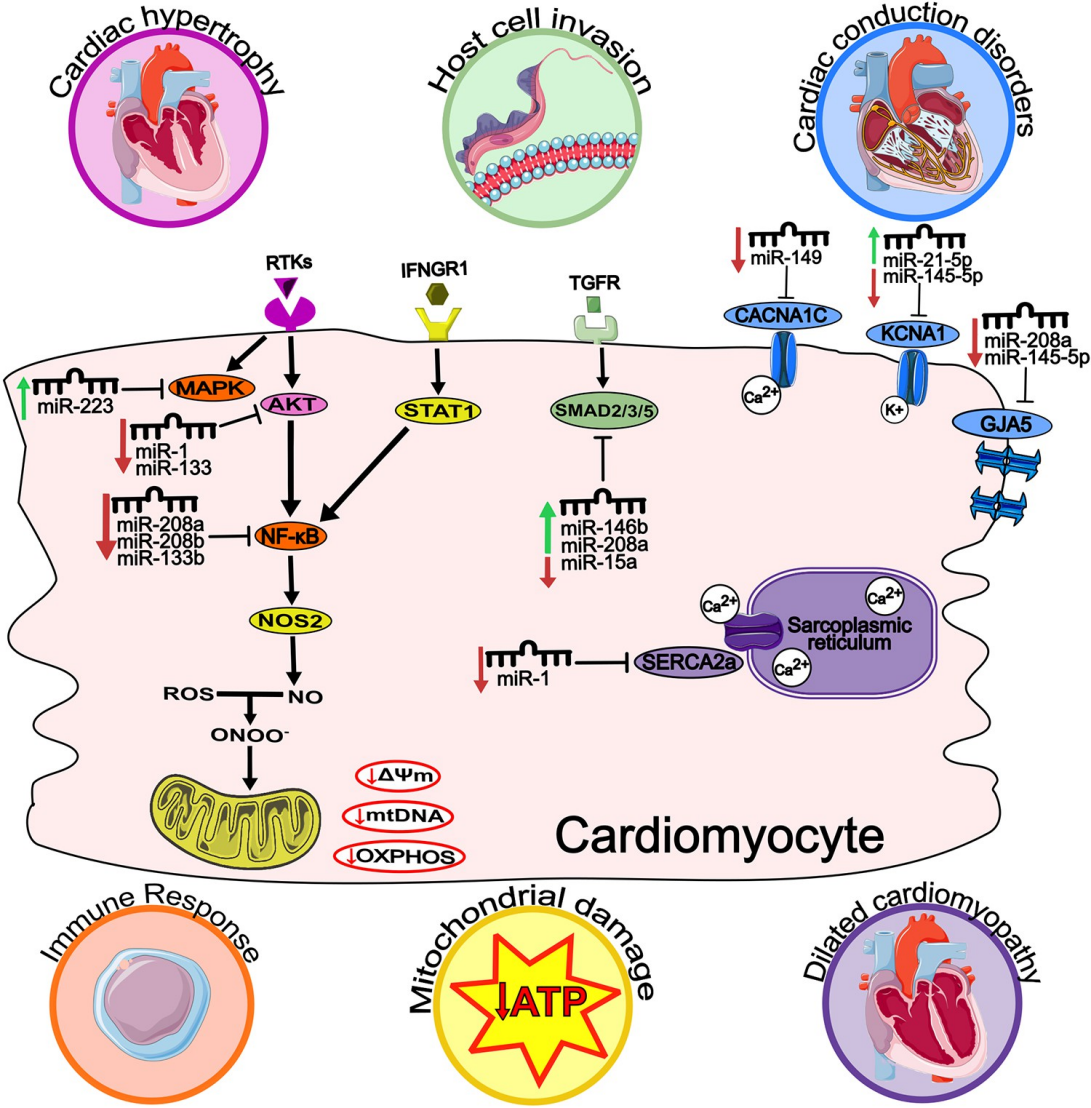
Probable main mechanisms involved in the pathophysiology of ChCM. RTKs: receptor tyrosine kinases; IFNGR1: interferon-gamma receptor 1; MAPK: mitogen-activated protein kinase; AKT: AKT serine/threonine kinase 1; STAT1: signal transducer and activator of transcription 1; NF-κB: nuclear factor kappa B; NOS2: nitric oxide synthase 2; ROS: reactive oxygen species; NO: nitric oxide; ONOO-: peroxynitrite; ΔΨm: mitochondrial membrane potential; mtDNA: mitochondrial DNA; OXPHOS: oxidative phosphorylation; CDND1: cyclin D1; TGFR: transforming growth factor beta receptor; SMAD2/3/5: SMAD family member 2, 3 and 5; SERCA2a: sarcoplasmic/endoplasmic reticulum Ca2+-ATPase; CACNA1C: calcium voltage-gated channel subunit alpha1 C; Ca2+: calcium ion; K+: potassium ion; KCNA1: potassium voltage-gated channel subfamily A member 1; GJA5: gap junction protein alpha 5. The hearts, receptors, ligands, ions channels, Trypanosoma, cell membrane, gap junctions, mitochondria, and lymphocyte cell in this figure have been modified from Servier Medical Art [28], licensed under a Creative Common Attribution 3.0 Generic License [29].

The miRNAs selected for this investigation have emerged as key players in the underlying mechanisms of ChCM (Fig 3), potentially serving as valuable disease biomarkers. Therefore, we conducted an exploration of the miRNA-target interactions of miR-21-5p, miR-145-5p, miR-146a-5p, miR-208a-3p, and miR-146b-5p to gain a deeper understanding of their involvement in the disease’s biological pathways. Through bioinformatics analysis, GO enrichment analysis revealed the roles of specific miRNAs and the enrichment of annotated terms associated with their gene targets. Among the predicted genes, 30 form a well-connected gene interaction network that plays a significant role in ChCM pathology (S1 Table). The detailed pathways information is presented in Fig 3.

**Fig 4:**
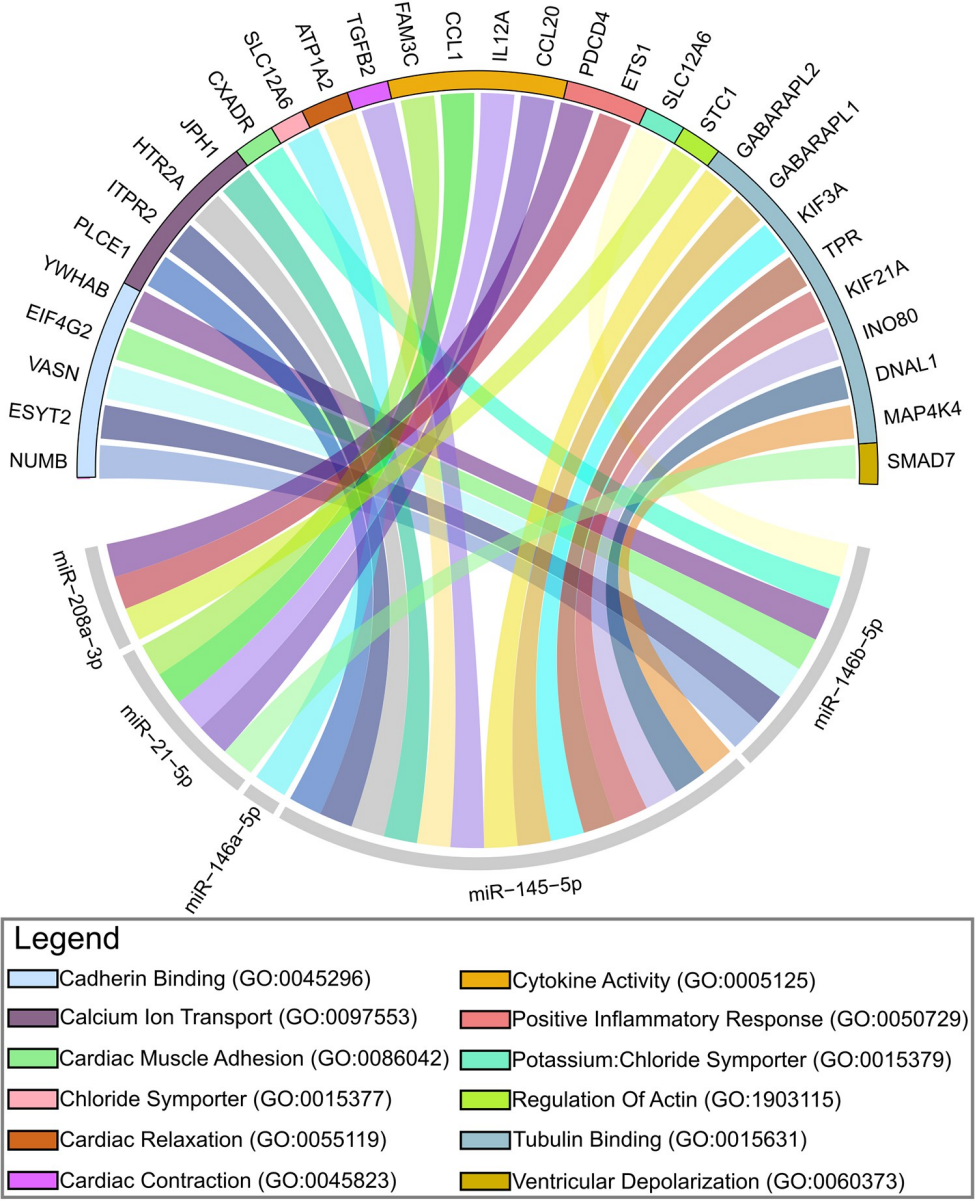
miRNA-target interaction considering biological process and molecular function in the cardiac and immunological context. The circular network shows miRNA and target interaction. Gene Ontology Biological Process (BM) and Molecular Function (MF) involved with the targets are color-coded at the top of the cycle. (GO: xxx = Gene Ontology entry number).

Our in silico analyses (Fig 4) demonstrated a significant relationship (*p* = 0.009) between miR-145-5p and its regulation of calcium transport (GO: 0097553). Lopez and colleagues [37] observed an aberrant increase in the Na+/Ca2+ Exchanger pathway in infected C57BL/6 mice when compared to the control group. The same pathway shows an experimental relationship with changes in ventricular depolarization in a study conducted by Santos-Miranda and colleagues [38]. In our enrichment analysis, miR-21-5p is linked to the “Ventricular Depolarization (GO: 0060373)” process (*p* = 0.03). Interestingly, in the 2 studies of this review miR-145-5p is up-regulated [23] and down-regulated [22], while miR-21-5p is up-regulated in all studies. Cardiac alterations related to the processes of “Cardiac Relaxation (GO: 0055119),” (*p* = 0.01) and “Cardiac Contraction (GO: 0045823),” (*p* = 0.01) are commonly described in different degrees in ChCM and may be related to the parasitic burden of *T*. *cruzi* [39]. On the other hand, 2 processes showed a relationship with the inflammatory response. miR-208a-3p is related to the “Inflammatory Response (GO:0050729)” (*p* = 0.02). Although the relationship between the process and miR-208a-3p is positive, the results of the analyzed studies show miRNA down-regulated in the cardiac phase. Conversely, miR-21-5p was also related to “Cytokine Activity (GO:0005125)” (*p* = 0.04). Many factors of the inflammatory response, such as the secretion of pro-inflammatory cytokines, activation of CD4+ and CD8+ T lymphocytes, and effector macrophages can contribute to the development and worsening of ChCM [40]. The processes and miRNAs “Tubulin Binding (GO: 0015631)” (*p* = 0.01), “Actin Regulation (GO: 1903115)” (*p* = 0.02), “Chloride Symporter (GO: 0015377)” (*p* = 0.04), “Cadherin Binding (GO: 0045296)” (*p* = 0.01), “Cardiac Muscle Adhesion (GO: 0086042)” (*p* = 0.03) did not show direct relationships with the disease according to our searches. Despite this, they are important processes related to cardiac physiology that need further investigation.

### Final considerations

In this review, despite the variations among the studies, miR-21, miR-146b, miR-146a, and miR-155 exhibited consistent expression patterns across multiple studies. In ChCM, a consistent up-regulation miR-21 was observed in cardiac tissue, serum, and plasma. Since miR-21 is associated with fibrosis and immune response, targeting miR-21 could be a promising therapeutic for ChCM [12,13,21,22]. The miR-146b was up-regulated during the acute phase of ChD specifically in cardiac tissue, and it seems to play a regulatory role in the interaction between the parasite and the host cell, suggesting its potential as a biomarker for therapeutic response [22,23,25]. Furthermore, both miR-155 and miR-146a are consistently up-regulated in plasma and cardiac tissue samples during the acute and chronic phases of ChD. However, miR-155 has an impact on oxidative stress in the hearts of individuals with ChCM, while circulating miR-146a is implicated in the inflammatory process and, therefore, could be a noninvasive biomarker for the early detection of ChD [12,20].

Regarding the down-regulation of miRNAs observed in the studies, only miR-145-5p was pointed out in the 2 studies [22,23]. Pioneering research in cardiac tissue samples from a C57BL6 model infected with *T*. *cruzi* showed a significant correlation between the levels of blood parasitemia and prolongation of the interval of depolarization and repolarization of the ventricles (QTc), a critical cardiac parameter related to cardiovascular damage. It was also observed that miRNA levels decline as infection progresses [22]. The level of miR-145-5p also decreases after combined Benznidazole and Pentoxifylline treatment in an infected rat cardiomyoblast, suggesting possible markers of pharmacotherapeutic response in ChD [23].

Finally, miR-208a showed decreasing results in a study conducted with human heart tissue from infected and non-infected patients [24]. Interestingly, the same miRNA was up-regulated in the blood plasma of ChCM patients [14].

However, in the indeterminate form of ChD (individuals with positive serology but without symptoms), miR-208a showed overexpression compared to uninfected patients. Of note, miR-208a has been reported as previously involved in cardiovascular processes such as hypertrophy, fibrosis, and arrhythmias, suggesting a target for further studies and a possible biomarker of clinical forms of the disease [14].

Despite these promising findings, it is important to acknowledge the limitations of the current studies included in this review. Further research is required to standardize approaches in studying miRNAs in ChCM, particularly through consortia efforts. Addressing crucial aspects such as disease duration, sample types, extraction and detection methods, and treatment protocols will enhance the reliability and reproducibility of miRNA-related investigations. Moreover, considering the pleiotropic effects of miRNAs, future studies need to develop standardized methodologies to accurately assess their overall impact on target mRNAs and biological processes.

In conclusion, our study highlights the crucial role of specific miRNAs in the pathophysiology of ChCM, particularly miR-21-5p, miR-145-5p, miR-146a-5p, miR-208a-3p, miR-146b-5p, and miR-155. These miRNAs demonstrate their involvement in key biological processes related to ChCM and hold potential as biomarkers for diagnosis, prognosis, and therapeutic targeting. Collaborative efforts, along with standardized approaches, will pave the way for identifying additional miRNAs and their multifaceted functions in different forms of ChD. Ultimately, these advancements will contribute to a deeper understanding of ChCM and facilitate the development of innovative strategies for its management and treatment.

**Key Learning Points**

- miR-21, miR-146b, miR-146a, and miR-155 play a key role in the development and progression of ChCM.
- miR-21 is presented as a promising therapeutic target.
- miR-146b, miR-155, miR-146a, and 208a are promising biomarkers in different stages of ChD.
- In silico analyses demonstrate the probable relationship of the described miRNAs with ChCM pathophysiological processes.
- Factors with disease stages, sample types, extraction, and detection methods can affect the reproducibility of miRNA studies.

**Five Key Papers**

1. Bern C. Chagas’ Disease. N Engl J Med. 2015;373:456–466. doi: 10.1056/NEJMra1410150
2. Laugier L, Ferreira LRP, Ferreira FM, Cabantous S, Frade AF, Nunes JP, et al. miRNAs may play a major role in the control of gene expression in key pathobiological processes in Chagas disease cardiomyopathy. PLoS Negl Trop Dis. 2020;14:e0008889. doi: 10.1371/journal.pntd.0008889
3. Nonaka CKV, Sampaio GL, de Aragão França L, Cavalcante BR, Silva KN, Khouri R, et al. Therapeutic miR-21 Silencing Reduces Cardiac Fibrosis and Modulates Inflammatory Response in Chronic Chagas Disease. Int J Mol Sci. 2021;22:3307. doi: 10.3390/ijms22073307
4. Ballinas-Verdugo MA, Jiménez-Ortega RF, Martínez-Martínez E, Rivas N, Contreras-López EA, Carbó R, et al. Circulating miR-146a as a possible candidate biomarker in the indeterminate phase of Chagas disease. Biol Res. 2021;54:21. doi: 10.1186/s40659-021-00345-3
5. Ferreira LRP, Frade AF, Santos RHB, Teixeira PC, Baron MA, Navarro IC, et al. MicroRNAs miR-1, miR-133a, miR-133b, miR-208a and miR-208b are dysregulated in Chronic Chagas disease Cardiomyopathy. Int J Cardiol. 2014;175:409–417. doi: 10.1016/j.ijcard.2014.05.019

## Supporting information

S1 Table Data from targets selected for analysis. (XLSX)
